# Etiology, clinical, and epidemiological characteristics of severe respiratory infection in people living with HIV

**DOI:** 10.1177/0956462419882587

**Published:** 2020-01-22

**Authors:** AC Pecego, RT Amâncio, DM Costa, FA Bozza, MM Siqueira, ML Oliveira, J Cerbino-Neto, A Japiassu

**Affiliations:** 1 Instituto Nacional de Infectologia – Fundação Oswaldo Cruz (Fiocruz) – Laboratório de Medicina Intensiva, Rio de Janeiro, Brazil; 2 Instituto D’Or de Pesquisa e Ensino, Rio de Janeiro, Brazil; 3 Instituto Oswaldo Cruz – Fiocruz – Laboratório de Vírus Respiratório, do IOC/FIOCRUZ, Rio de Janeiro, Brazil; 4 Laboratório de Pesquisa em Imunização e Vigilância em Saúde, Rio de Janeiro, Brazil

**Keywords:** Human immunodeficiency virus, viral disease, bacterial disease, viral disease

## Abstract

People living with HIV (PLWH) are more prone to severe respiratory infections. We used the severe acute respiratory infection (SARI) definition to describe the etiology, clinical, and epidemiological characteristics in this population. This was a prospective observational study including PLWH hospitalized with fever and cough. Those with symptom onset up to 10 days were classified as severe acute respiratory infection and 11–30 days as non-severe acute respiratory infection. Blood, urine samples and nasopharyngeal swabs were collected. Data were extracted from patient charts during their hospital stay. Forty-nine patients were included, median CD4 cell count: 80 cells/mm^3^, median time since HIV diagnosis and hospital admission: 84 months and 80% were antiretroviral therapy exposed. Twenty-seven patients were classified as SARI. Etiology was identified in 69%, 47% were polymicrobial. Respiratory virus (9 SARI vs. 13 non-SARI), bacteria (5 SARI vs. 4 non-SARI), *Mycobacterium tuberculosis* (6 SARI group vs. 7 non-SARI group), *Pneumocystis jirovecii* (4 SARI vs. 1 non-SARI), *Cryptococcus neoformans* (1 SARI vs. 3 non-SARI), and influenza A (1 SARI vs. 2 non-SARI). Dyspnea was statistically more prevalent in SARI (78% vs. 36%, *p* = 0.011) but the risk of death was higher in the non-SARI (4% vs. 36%, *p* = 0.0067). In the severely immunocompromised PLWH, severe acute respiratory infection can be caused by multiple pathogens and codetection is a common feature.

## Background

People living with HIV (PLWH) are highly susceptible to respiratory infections.^1^ Even in the antiretroviral therapy (ART) era, tuberculosis (TB) has fallen disproportionately among seropositive patients^2^ and pneumonia remains five times more common among this population, despite achieving CD4 cell counts above 500 cells/mm^3^.^3^ PLWH are also at increased risk for poor influenza outcomes, which may be particularly risky for those living in countries with limited resources.^4–6^

After the recent influenza A (H1N1, H5N1, and H7N9) and the Middle East Respiratory Virus (MERS-CoV) outbreaks, the WHO is encouraging and supporting countries to strengthen surveillance on severe acute respiratory infections (SARI)^7^ but with limited information on PLWH regarding etiology and prognosis, despite their increased risk for respiratory infections and adverse outcomes.^8–10^ So, in this study, we described how SARI is represented, according to clinical presentation, epidemiology and etiology in a population of PLWH with respiratory infection residing in a high-prevalence TB area.

## Patients and methods

### Study design

A prospective observational study was conducted at the Instituto Nacional de Infectologia Evandro Chagas (INI-Fiocruz), Rio de Janeiro, Brazil, from May 2012 to December 2013. INI-Fiocruz is a national reference center for infectious diseases and has been a reference center for care, research, and training related to HIV/AIDS since 1986. The AIDS program at INI is one of the largest providers of primary, specialty, and tertiary care for PLWH patients in Rio de Janeiro State with a cohort of about 4000 patients in active follow-up.

### Patients

**Figure 1.**
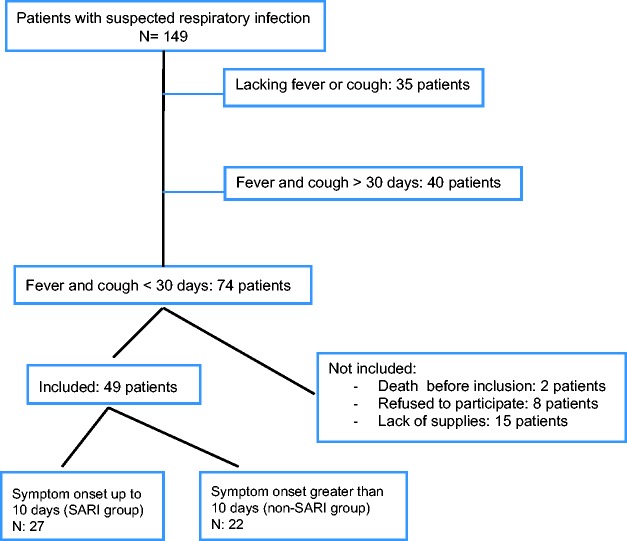
Flowchart of inclusion.

We conducted surveillance of every HIV-positive patient hospitalized during the study period above. Every Monday, Wednesday, and Friday we carried out reviews of inpatient medical records and all PLWH hospitalized with respiratory symptoms were screened by the principal investigator, a physician specialized in infectious diseases. In the presence of fever and/or cough and onset of symptoms for less than 30 days, the patient was invited to participate in the study, which involved the collection of clinical material as detailed below in the sample collection and processing section. Patients were excluded if symptoms occurred more than 30 days ago or if no fever and/or no cough were reported by the patient. Patients were classified as SARI if the onset of symptoms was ≤10 days, according to WHO global standards for SARI definition.^7^ Those with symptom onset greater than 10 days but up to 30 days prior were considered as non-SARI patients (Figure 1). Participants were screened by a physician regarding respiratory viral symptoms such as myalgia, diarrhea, arthralgia, coryza, and odynophagia. Data on dyspnea, respiratory frequency, and peripheral oxygen saturation (SpO_2_) using pulse oximetry were collected by a respiratory therapist. Patients were considered to have dyspnea if rated ≥5 on an ascending scale from 0 to 10.

### Data collection

Clinical and epidemiological data were collected during hospitalization using standardized forms, until hospital discharge or death (Table 1). Variables of interest were age, gender, time since HIV diagnosis based on the first HIV-positive result, CD4 nadir cell count, and CD4 cell count at hospital presentation or up to 2 months before, having had a previous TB diagnosis (pulmonary or disseminated) or any other previous opportunistic infection. Viral load was considered to be undetectable if less than 400 copies/mL, and we considered being ART exposed if patient ever used ART regardless of adherence. Prophylaxis of respiratory infection was recorded, such as *Pneumocystis jirovecii* pneumonia (PJP) prophylaxis and pneumococcal and influenza vaccines. Data about the respiratory infection included leucocyte count, C-reactive protein, pulse oximetry, and CRB-65 and the combined CRB-65 + O_2_ score at admission. Outcome variables of interest were: intensive care unit (ICU) admission, non-invasive ventilation use beginning within 72 h of hospital admission and death.

**Table 1.** Clinical and epidemiological characteristics of patients with less than 10 days since symptom onset (SARI) vs patients with more than 10 days since symptom onset (non-SARI).

Variables	Total*N* = 49	SARI*N* = 27	Non-SARI*N* = 22	*P* value
Age	38 (34–43)	38 (29–48)	40 (27–53)	0.65
Gender (male)	27 (55%)	15 (56%)	11 (50%)	0.77
Time since HIV diagnosis^a^	84 (13–141)	92 (11–145)	84 (12–135)	0.10
CD4 nadir	58 (9–156)	94 (22–240)	51 (6–149)	0.61
CD4 (cells/mm^3^)^b^	80 (24–274)	107 (25–372)	81 (6–259)	0.82
ART exposure	38 (80%)	20 (74%)	18 (82%)	0.73
VL < 400 copies/ml^c^	12 (25%)	6 (22%)	6 (27%)	0.74
Previous TB	24 (49%)	13 (48%)	11 (50%)	1.00
Previous OI	18 (37%)	9 (33%)	9 (41%)	0.76
PJP prophylaxis	7 (14%)	4 (15%)	3 (14%)	1.00
Influenza vaccine	9 (18%)	7 (26%)	2 (9%)	0.16
Pneumo23 vaccine	8 (16%)	4 (15%)	3 (14%)	1.00
SatO_2_ < 90%	15 (31%)	8 (30%)	7 (32%)	1.00
Multilobar X-ray changes	31 (63%)	16 (59%)	15 (68%)	0.38
Leucocyte count (cells/mm^3^)	7330 (4570–10,930)	8700 (5400–11.730)	7100 (4250–12.995)	0.86
C-reactive protein	12 (8.7–20.4)	12 (7–22)	13 (9–20)	0.63
ICU admission	17 (35%)	8 (30%)	9 (41%)	0.55
NIV < 72 h	33 (67%)	17 (63%)	12 (55%)	0.57
Etiological Identification	34 (69%)	18 (63%)	16 (73%)	0.76
Death	9 (18%)	1 (4%)	8 (36%)	0.0067

VL: viral load; ART: antiretroviral therapy; PJP: *Pneumocystis jirovecii* pneumonia; TB: tuberculosis; MV: mechanical ventilation; NIV: non-invasive ventilation; OI: opportunistic infection; SARI; severe acute respiratory infection.

The results are expressed as percentile for categorical variables and as the medium values and the interquartile range for continuous variables.

a Time expressed in months.

b CD4 cell count available for 47 patients.

c VL—viral load available for 44 patients.

Microbiologically-confirmed pneumonia was defined as fever (axillary temperature) ≥38°C, (new) pulmonary infiltrate on chest X-ray or computed tomography scan plus an etiological agent identified through the tests mentioned below.

### Sample collection and processing

For every patient included we collected blood cultures for bacteria, fungi, and mycobacteria processed with the automated BactAlert® system (Healthcare-bio Merieux), urine samples for urinary *Streptococcus pneumoniae* and *Legionella pneumophila* serogroup 1 testing (Binaxnow kit®) and triple respiratory swab (nasopharyngeal, one for each nostril and one for posterior pharynges). Respiratory swabs were tested for respiratory syncytial virus (RSV), adenovirus (Adeno), influenza A (FluA), parainfluenza 1–3 (PIV 1–3), human-metapneumovirus (HMPV), and rhinovirus (RV) using rt-PCR. Probes were labeled at the 5′ terminus with 6 carboxyfluorescein (FAM) and with quencher Blackhole-1 dT (Biosearch Technologies, Inc., Novato, CA).

The tests were done following Centers for Disease Control and Prevention (CDC) guidance. Sputum or induced sputum and bronchoalveolar fluid were tested for fungus and *Mycobacterium* spp. under direct visualization followed by inoculation into Sabouraud culture medium for fungi and BACTEC® MGIT™ 960 and Lowenstein–Jensen for mycobacterial culture. *Mycobacterium* spp. identification was performed under conventional techniques (Kent and Kubica, 1985, CDC Atlanta). All clinical samples were collected up to 72 h after hospital admission except for *P. jirovecii* detection which was done through staining and indirect immunofluorescence assay (IFL) on bronchoalveolar samples, which were performed only in mechanically-ventilated patients.

### Statistical analysis

We conducted a descriptive analysis of the convenience sample from the studied population. Categorical variables were presented as proportion and continuous variables summarized as median and interquartile ranges for SARI and non-SARI patients. We compared the distribution of continuous variables by using U or Mann–Whitney test, and categorical variables by using the Chi-squared or Fisher’s exact test when appropriate at univariate analysis. The analyses were processed using Statistical Package for Social Science (SPSS) and GraphPad Prism 3.0 for Windows (GraphPad Software, San Diego, CA, USA). Statistical significance was considered whenever *p* value was less than 0.05.

## Results

A total of 149 patients were screened and 74 patients met our inclusion criteria. Among those, 8 patients refused to participate because of discomfort and pain during the nasal swab collection and 15 patients could not be included due to temporary lack of supplies. A total of 49 patients with fever and cough and symptoms lasting up to 30 days were included (Figure 1), of which 27 (55%) met the SARI case definition.

A profound and prolonged immunosuppression is reflected by the median nadir lymphocyte CD4 cell count (58 cells/mm^3^) and at hospital admission (80 cells/mm^3^) and time since HIV diagnosis with a median of 84 months, except for five patients with recent HIV diagnosis (in the previous three months). This finding is supported by the fact that 38 (80%) patients were ART exposed, but only 12 (25%) had an undetectable viral load. Prevention of respiratory infection was observed in only 7 (14%) patients taking prophylactic sulfamethozaxole–trimethoprim for PJP, 7 patients (14%) who previously received *S. pneumoniae* vaccine and 9 (18%) received influenza seasonal vaccine. About half of the patients had a previous history of TB and 18 (37%) had a history of other opportunistic infections.

Pneumonia was confirmed in 46 (94%) patients and 31 (63%) had X-rays with multilobar infiltrates. In the remaining three patients, the chest X-ray was normal and CT scan could not be done (data not shown). Pulse oximetry showed that 15 patients (30%) had SpO_2_ less than 90% and non-invasive ventilation was initiated in 67% of the patients in the first 72 h of admission. Twenty (41%) patients were classified as CRB-65 = 0 points, 19 patients classified as CRB-65 = 1 (39%) point, 9 (18%) as CRB-65 = 2 points and 1 as CRB-65 = 3 points. Although 39 patients had CRB-65 0–1, the majority required non-invasive ventilation (NIV) in the first 72 h of hospitalization and 25 had chest X-rays with multilobar infiltrates. The mortality risk for those 10 patients (20.4%) with CRB-65 2–3 points was 4.5 higher (95% confidence interval [CI]: 0.94–21.92) compared to CRB-65 0–1 points, *p* value = 0.069, with sensitivity (S) and specificity (Sp) of 85% and 44% and positive predictive value (PPV) and negative predictive value (NPV) of 87% and 40%, respectively. When combined with pulse oximetry, 15 patients (30.6%) had CRB65+O_2_ of 2–4 points and a 10.5 higher risk of dying (95% CI: 1.87–59.1), *p* value = 0.0051 with a S:75% and Sp:78% and PPV: 94% and NPV: 42% (data not shown). In total, nine patients (18.6%) died during hospitalization.

The median time between symptom onset and hospital admission was 10 days (6–20). According to SARI classification, we saw that clinical and epidemiological characteristics were evenly distributed with no statistical difference between SARI and non-SARI patients, except for mortality rate, which was significantly more prevalent among non-SARI patients (Table 1).

### Symptoms according to SARI classification

**Figure 2.**
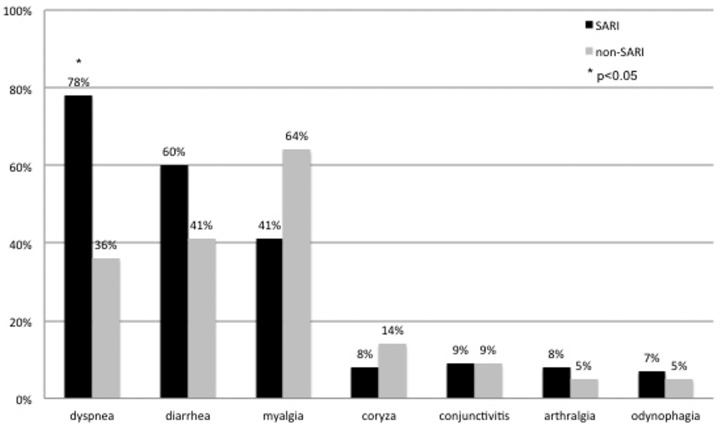
Symptoms (%) according to SARI and non-SARI classification.SARI: severe acute respiratory infection.

Symptoms related to respiratory viral infection were analyzed among SARI and non-SARI patients (Figure 2). Fever and cough were present in 100% of cases because they were used as inclusion criteria. Dyspnea was the only symptom that was statistically more prevalent in the SARI subgroup, *p* = 0.011 (Figure 2). Arthralgia, conjunctivitis, odynophagia, and coryza were all infrequent in both subgroups.

### Etiology of respiratory infection according to SARI classification

Microbiological confirmation was possible in 34 (69%) patients and 16 (47%) patients had polymicrobial infections (Table 2). Viral pathogens were the most common agents, occurring in 17 (35%) patients with 22 isolates. RV was the most common virus among SARI (4; 15%) and non-SARI (7; 32%) patients, followed by RSV in (5; 10%); the majority (4/5) in the non-SARI group, influenza A in 3 (6%), HMPV in 2 (4%), and parainfluenza-2 in 1 (2%) patient. All three cases of influenza A happened in non-influenza-vaccinated patients, but since the vaccination was infrequent, we could not explore its impact. Influenza A(pdm09)H1N1 was confirmed in one patient classified as SARI, and the other two influenza viruses were identified among patients whose symptom onset was greater than 10 days ago, both seasonal AH3N2 strains. The two isolates of HMPV and the only PIV-2 were identified in patients with SARI.

**Table 2.** Etiology and percentage of codetection with other pathogens among SARI and non-SARI patients.

Etiology	Isolates	SARI	Codetection	Non-SARI	Codetection
Viral detection	22	9		13	
Rhinovirus	11	4	50%	7	43%
HMPV	2	2	50%	0	–
RSV	5	1	50%	4	100%
PIV-2	1	1	–	0	–
Influenza A	2	0	–	2	100%
Influenza A(pdm09)H1N1	1	1	100%	0	
Other pathogens					
*Mycobacterium tuberculosis*	13	6	67%	7	43%
*Streptococcus pneumoniae*	7	4	50%	3	100%
*Pneumocystis jirovecii*	5	4	75%	1	–
Cryptococcus neoformans	4	1	100%	3	75%
*Legionella pneumophila*	1	1	–	0	–
*Pseudomonas aeruginosa*	1	0	–	1	100%
Total	53	25		28	
% codetection	33%	26%		41%	

PIV: parainfluenza-2; HMPV: human-metapneumovirus; RSV: respiratory syncytial virus; SARI: severe acute respiratory infection.

*Mycobacterium tuberculosis* (Mtb) was the most common bacterial pathogen identified in 13 (27%) patients (Table 2). Among the 24 patients (49%) who had been previously diagnosed with TB, 8/24 (33%) were readmitted with Mtb infection the majority in the non-SARI group (7/8 patients, 88%).

*S. pneumoniae* was evenly distributed among SARI (four isolates) and non-SARI (three isolates), but PJP was more frequent in the SARI group, while *Cryptococcus neoformans* was more common in the non-SARI group (Table 2).

Among polymicrobial infections, 13 patients had 2 pathogens and 3 patients had 3 or more pathogens identified. Polymicrobial infection was more frequent in the non-SARI group, 9 (41%) vs. 7 (26%), but with no statistical difference. No single agent was significantly more prevalent according to this classification.

The main agents associated with severity and ICU admission were PJP in 80% (4/5); *Cryptococcus neoformans* in 75% (3/4), *S. pneumoniae* in 43% (3/7), and *Pseudomonas aeruginosa* in 100% (1/1) of the cases. Infections were polymicrobial in 41% vs. 30% of the patients being admitted to the ICU (*p* = 0.52) and in 44% of the patients who died vs. 25% among survivors (*p* = 0.25).

## Discussion

We found that PLWH hospitalized with fever and cough, with less than 30 days of symptoms were mostly young, with severe and prolonged immunosuppressed caused by HIV infection. There were no clinical or epidemiological aspects that could distinguish the classification of SARI and non-SARI. In terms of etiology, SARI was represented by a broad range of infectious agents in this population and much of the lung infections were polymicrobial. In one-third of the cases, we found viruses in nasopharyngeal samples and two out of three influenza viruses were detected in non-SARI patients.

Our findings highlight the importance of viral pathogens in community-acquired respiratory infections: viruses were implicated in 35% of respiratory symptoms in PLWH and RV was the most common one. This may be due to the fact that the RV is present throughout the year regardless of specific seasonality.^11,12^ Although being historically related to the common cold and to mild respiratory illness,^13–15^ recent data suggest that RV can be related to more severe cases.^16^ According to Cohen et al. it was the principal agent of SARI during a hospital surveillance period 2009–2012 in South Africa, where 74% were PLWH^9^ and also an important agent in elderly hospitalized with SARI in Chile^17^ as well as the causal agent of more severe cases requiring ICU treatment, in co-detection with *S. pneumoniae.*^18^ Most of our cases were found in co-detection with another agent suggesting that RV may contribute to more severe cases but the possibility of only being a bystander cannot be rule out in this context. In accordance to our findings, HMPV remains an infrequent cause of SARI even in PLWH.^19^

Apart from viral detection, *S. pneumoniae* is reemphasized as the main bacterial pathogen found in patients with CAP who presented with SARI, regardless of the CD4 lymphocyte count as shown by López-Aldeguer et al.^20^ Another important aspect of our study was the fact this pyogenic bacterium was found in patients with late onset of symptoms (non-SARI) and three out of seven patients presented with bacteremia and all three of them died, a feature often correlated with poor prognosis.^21^ *L. pneumophila* remains an uncommon cause of community-acquired lung infection but we acknowledge that some cases might have been missed since the urinary antigen test can only detect type 1 infections and up to 14 days of onset.^20,22^

We found a high frequency of Mtb in patients with few days of symptoms and in a population with a previous history of pulmonary or disseminated TB, suggesting that prolonged immunosuppression may reactivate the bacillus.^22–24^ Our findings are in agreement with Coelho et al. who showed that TB was the most common opportunistic infection in the total cohort followed at our center. In high TB prevalence settings, such as the one observed in our context, this diagnosis should be pursued in PLWH regardless of symptom duration.^25^

Another important aspect of our findings was to emphasize the polymicrobial aspect of respiratory infections in this population. Co-detection of multiple pathogens can occur in the context of HIV infection^20,25,26^ but also in the general adult population as recently demonstrated by Karhu et al. (39% of severe acquired pneumonia patients) and in children with SARI.^26–28^

In our sample, coinfection could be associated with death (44% vs. 25%, *p* = 0.25) but did not reach statistical significance, probably due to the small sample size.

Whether the presence of multiple pathogens leads to a more severe presentation with increased risk of poor outcomes is still unclear.^29^ When looking at coinfection involving bacteria plus a respiratory virus, mortality seems to be higher in the bacterial coinfection subgroup,^30,31^ as shown by Walaza et al. with TB and influenza and by Crotty et al. in pneumonia patients.^32,33^ But these findings may not be applied to viral–viral coinfection.^34^

Since SARI and non-SARI were similar according to clinical and epidemiological aspects, the delay between symptom onset and the demand for care (non-SARI group) could have contributed to the higher mortality observed in our findings, as shown previously by several authors.^6,35,36^ Although 39 patients had CRB-65 of 0–1 points and could have been sent home with oral antibiotic therapy, the majority required NIV in the first 72 h of hospital admission and 63% had X-rays with multilobar infiltrates, another predictor of unfavorable prognosis. Similar findings were found by Almeida et al. in 49 PLWH admitted with pneumonia in an emergency department with a median CRB65 score lower then HIV-negative patients but which were also associated with a higher risk of mortality.^37^ In the paper of Yone et al., 54 out 62 PLWH admitted with community acquired pneumonia (CAP) had CRB65 of 0–1.^38^ In our study, adding the pulse oximetry to CRB-65 score, as suggested by the Consensur II, the Latin America Pneumonia working Group, increased the ability to confidently predict the 30-day in-hospital mortality.^39^

In the context of SARI surveillance target at influenza, a possible explanation for our late detection relies on the fact that immunosuppressed and critically ill patients there seems to be prolonged viral shedding.^40^ The median CD4 cell count in this study population was 80 (24–274) cells/mm^3^ and the proportion of patients with undetectable viral load was 25% despite 80% being exposed to ART. Compared to the whole cohort followed at our center for the period 2012–2013, where 69.5% were male and the median age was 42.5 years, the median CD4 cell count was higher (542 cells/mm^3^), and so was the proportion who had undetectable HIV viral load (69.5%) and ever being exposed to ART (90.8%).^41^ This shows that the population in this study is severely immunocompromised and found it harder to adhere to ART at the time. We observed two cases of influenza in the non-SARI group, respectively 20 and 30 days after symptom onset, and both patients developed respiratory failure that led to death (data not shown). This highlights the fact that immunosuppressed PLWH with respiratory symptoms may not seek hospital attention in time to be captured by SARI surveillance.

Finally, regarding symptom frequency, we found a low percentage of sore throat, runny nose and conjunctivitis and a high percentage of dyspnea, diarrhea, and myalgia. In fact, dyspnea was the only symptom more prevalent in the SARI group (*p* < 0.05). Although gastrointestinal involvement may have been influenced by the advanced immunodeficiency syndrome, other authors studying the symptoms of influenza in less-immunocompromised PLWH reached similar results.^42,43^ It is important to note that our study tried to address viral symptoms in a context of multiple pathogens. On one hand, it is difficult to assign a symptom to a specific virus or bacteria but on the other hand, it may reflect what actually happens in clinical practice, because as already highlighted above, a significant percentage of respiratory infections, especially in PLWH are of mixed type.

Our study has limitations. The small sample size conducted at a single center does not allow generalization of the results and the detection of respiratory viruses from the lower respiratory tract would have been more representative of the etiology of respiratory infection. Patients with advanced HIV infection may have multiple infections at the same time and an overlap of signs and symptoms is frequent, so the need for hospitalization may not be attributed exclusively to the respiratory infection *per se.* All cases in this study occurred in a 19-month period at a single institution, and viral epidemiology at this site and during this timeframe may not be representative of all seasons and locations. Finally, due to the constraints of the respiratory viral kit used, we may not have identified all possible and important viral infections such as adenovirus, coronavirus and bocavirus and PCR technique may detect a virus that is representative of several conditions such as the true agent itself, a bystander or facilitating bacterial superinfection.^44^

## Conclusion

In severely immunocompromised PLWH, SARI can be represented by a wide variety of respiratory pathogens and the codetection of multiple pathogens is common. Viruses must be considered as a common cause of respiratory infection either alone or in combination with other pathogens and in scenarios where TB is endemic, this agent should be pursued regardless of cough duration.

## Data Availability

De-identified data and materials are available on a case-by-case basis. Please contact the corresponding author with requests.

## References

[1] Segal LN Methe BA Nolan A, et al HIV-1 and bacterial pneumonia in the era of antiretroviral therapy. Proc Am Thorac Soc 2011; 8: 282–287. 21653529 10.1513/pats.201006-044WR PMC3132786

[2] Pacheco AG Durovni B Cavalcante SC, et al AIDS-related tuberculosis in Rio de Janeiro, Brazil. PLoS ONE 2008; 3: e3132. 18781195 10.1371/journal.pone.0003132 PMC2518952

[3] Sogaard OS Lohse N Gerstoft J, et al Hospitalization for pneumonia among individuals with and without HIV infection, 1995–2007: a Danish population‐based, nationwide cohort study. Clin Infect Dis 2008; 47: 1345–1353. 18834317 10.1086/592692

[4] Tempia S Walaza S Viboud C, et al Mortality associated with seasonal and pandemic influenza and respiratory syncytial virus among children <5 years of age in a high HIV prevalence setting—South Africa, 1998-2009. Clin Infect Dis 2014; 58: 1241–1249. 24567249 10.1093/cid/ciu095 PMC9096151

[5] Tempia S Walaza S Viboud C, et al Deaths associated with respiratory syncytial and influenza viruses among persons ≥5 years of age in HIV-prevalent area, South Africa, 1998–2009^1^. Emerg Infect Dis 2015; 21: 600–608. 25811455 10.3201/eid2104.141033 PMC4378466

[6] Cohen C Moyes J Tempia S, et al Mortality amongst patients with influenza-associated severe acute respiratory illness, South Africa, 2009-2013. PloS One 2015; 10: e0118884. 25786103 10.1371/journal.pone.0118884 PMC4365037

[7] WHO|WHO Global Epidemiological Surveillance Standards for Influenza. WHO 2014. http://www.who.int/influenza/resources/documents/influenza_surveillance_manual/en/ (accessed 17 April 2014).

[8] Wansaula Z Olsen SJ Casal MG, et al Surveillance for severe acute respiratory infections in Southern Arizona, 2010-2014. Influenza Other Respi Viruses 2016; 10: 161–169. 10.1111/irv.12360 PMC4814863 26590069

[9] Cohen C Walaza S Moyes J, et al Epidemiology of severe acute respiratory illness (SARI) among adults and children aged ≥5 years in a high HIV-prevalence setting, 2009–2012. Plos One 2015; 10: e0117716. 25706880 10.1371/journal.pone.0117716 PMC4337909

[10] Pretorius MA Tempia S Walaza S, et al The role of influenza, RSV and other common respiratory viruses in severe acute respiratory infections and influenza-like illness in a population with a high HIV sero-prevalence, South Africa 2012–2015. J Clin Virol 2016; 75: 21–26. 26741826 10.1016/j.jcv.2015.12.004 PMC5712432

[11] Njouom R Yekwa EL Cappy P, et al Viral etiology of influenza-like illnesses in Cameroon, January-December 2009. J Infect Dis 2012; 206: S29–S35. 23169968 10.1093/infdis/jis573 PMC7107314

[12] Monto AS. The seasonality of rhinovirus infections and its implications for clinical recognition. Clin Ther 2002; 24: 1987–1997. 12581541 10.1016/S0149-2918(02)80093-5 PMC7133757

[13] Lieberman D Shimoni A Shemer-Avni Y, et al Respiratory viruses in adults with community-acquired pneumonia. Chest 2010; 138: 811–816. 20363845 10.1378/chest.09-2717 PMC7094496

[14] Johansson N Kalin M Tiveljung‐Lindell A, et al Etiology of community‐acquired pneumonia: increased microbiological yield with new diagnostic methods. Clin Infect Dis 2010; 50: 202–209. 20014950 10.1086/648678 PMC7107844

[15] For Beijing Network for Adult Community-Acquired Pneumonia (BNACAP), Qu J-X Gu L Pu Z-H, et al Viral etiology of community-acquired pneumonia among adolescents and adults with mild or moderate severity and its relation to age and severity. BMC Infect Dis 2015; 15: 1--7. 10.1186/s12879-015-0808-0 PMC4342096 25812108

[16] Jain S Self WH Wunderink RG, et al Community-acquired pneumonia requiring hospitalization among U.S. adults. N Engl J Med 2015; 373: 415–427. 26172429 10.1056/NEJMoa1500245 PMC4728150

[17] Fica A Dabanch J Andrade W, et al Clinical relevance of rhinovirus infections among adult hospitalized patients. Braz J Infect Dis 2015; 19: 118–124. 25523079 10.1016/j.bjid.2014.10.003 PMC7185615

[18] Choi S-H Hong S-B Ko G-B, et al Viral infection in patients with severe pneumonia requiring intensive care unit admission. Am J Respir Crit Care Med 2012; 186: 325–332. 22700859 10.1164/rccm.201112-2240OC

[19] Groome MJ Moyes J Cohen C, et al Human metapneumovirus-associated severe acute respiratory illness hospitalisation in HIV-infected and HIV-uninfected South African children and adults. J Clin Virol 2015; 69: 125–132. 26209394 10.1016/j.jcv.2015.06.089 PMC9134797

[20] López-Aldeguer J Iribarren JA Valencia E, et al Outcomes in HIV-infected patients admitted due to pandemic influenza. Enfermedades Infecc Microbiol Clínica 2012; 30: 608–612. 10.1016/j.eimc.2012.02.007 22459686

[21] Hull MW Phillips P Montaner J. Changing global epidemiology of pulmonary manifestations of HIV/AIDS. Chest 2008; 134: 1287–1298. 19059959 10.1378/chest.08-0364

[22] Mayaud C Parrot A Cadranel J. Pyogenic bacterial lower respiratory tract infection in human immunodeficiency virus-infected patients. Eur Respir J 2002; 20: 28S–39s. 10.1183/09031936.02.00400602 12168745

[23] Kwan CK Ernst JD. HIV and tuberculosis: a deadly human syndemic. Clin Microbiol Rev 2011; 24: 351–376. 21482729 10.1128/CMR.00042-10 PMC3122491

[24] Pawlowski A Jansson M Sköld M, et al Tuberculosis and HIV co-infection. PLoS Pathog 2012; 8: e1002464. 22363214 10.1371/journal.ppat.1002464 PMC3280977

[25] Saraceni V Cohn S Cavalcante SC, et al Prevalent tuberculosis at HIV diagnosis in Rio de Janeiro, Brazil: the TB/HIV in Rio (THRio) cohort. JAIDS J Acquir Immune Defic Syndr 2014; 67: 98–101. 24933097 10.1097/QAI.0000000000000247 PMC4497547

[26] Garbino J, Mossdorf E, Inoubli S, et al. Respiratory viruses in HIV-infected patients with suspected respiratory opportunistic infection. *AIDS* 2008; 22: 701--705. 10.1097/QAD.0b013e3282f470ac 18356599

[27] Karhu J Ala-Kokko TI Vuorinen T, et al Lower respiratory tract virus findings in mechanically ventilated patients with severe community-acquired pneumonia. Clin Infect Dis 2014; 59: 62–70. 24729498 10.1093/cid/ciu237 PMC4305142

[28] Nascimento-Carvalho CM Ribeiro CT Cardoso MRA, et al The role of respiratory viral infections among children hospitalized for community-acquired pneumonia in a developing country. Pediatr Infect Dis J 2008; 27: 939–941. 18756190 10.1097/INF.0b013e3181723751

[29] Damasio GAC Pereira LA Moreira SDR, et al Does virus-bacteria coinfection increase the clinical severity of acute respiratory infection?: Virus-bacteria coinfection and respiratory infection. J Med Virol 2015; 87: 1456–1461. 25976175 10.1002/jmv.24210 PMC7166438

[30] Kim H-C Choi S-H Huh J-W, et al Different pattern of viral infections and clinical outcomes in patient with acute exacerbation of chronic obstructive pulmonary disease and chronic obstructive pulmonary disease with pneumonia: respiratory viral infections in COPD patients. J Med Virol 2016; 88: 2092–2099. 27187664 10.1002/jmv.24577 PMC7166762

[31] Cebey-Lopez M Herberg J Pardo-Seco J, et al Does viral co-infection influence the severity of acute respiratory infection in children? PloS One 2016; 11: e0152481. 27096199 10.1371/journal.pone.0152481 PMC4838299

[32] Walaza S Tempia S Dawood H, et al Influenza virus infection is associated with increased risk of death amongst patients hospitalized with confirmed pulmonary tuberculosis in South Africa, 2010–2011. BMC Infect Dis 2015; 15. doi:10.1186/s12879-015-0746-x. 10.1186/s12879-015-0746-x PMC4316613 25623944

[33] Crotty MP Meyers S Hampton N, et al Epidemiology, co-infections, and outcomes of viral pneumonia in adults: an observational cohort study. Medicine (Baltimore) 2015; 94: e2332. 26683973 10.1097/MD.0000000000002332 PMC5058945

[34] Asner SA Science ME Tran D, et al Clinical disease severity of respiratory viral co-infection versus single viral infection: a systematic review and meta-analysis. PLoS ONE 2014; 9: e99392. 24932493 10.1371/journal.pone.0099392 PMC4059637

[35] Ormsby CE de la Rosa-Zamboni D Vázquez-Pérez J, et al Severe 2009 pandemic influenza A (H1N1) infection and increased mortality in patients with late and advanced HIV disease. AIDS 2011; 25: 435–439. 21139486 10.1097/QAD.0b013e3283434844

[36] Lynfield R Davey R Dwyer DE, et al Outcomes of influenza A(H1N1)pdm09 virus infection: results from two international cohort studies. PLoS ONE 2014; 9: e101785. 25004134 10.1371/journal.pone.0101785 PMC4086938

[37] Almeida A Almeida AR Castelo Branco S, et al CURB-65 and other markers of illness severity in community-acquired pneumonia among HIV-positive patients. Int J STD AIDS 2016; 27: 998–1004. 26394997 10.1177/0956462415605232

[38] Yone EWP Balkissou AD Kengne AP, et al Influence of HIV infection on the clinical presentation and outcome of adults with acute community-acquired pneumonia in Yaounde, Cameroon: a retrospective hospital-based study. BMC Pulm Med 2012; 12: 1--6. 10.1186/1471-2466-12-46 PMC3495717 22935579

[39] Bantar C Curcio D Jasovich A, et al. Neumonía aguda adquirida en la comunidad en adultos: Actualización de los lineamientos para el tratamiento antimicrobiano inicial basado en la evidencia local del Grupo de Trabajo de Sudamérica (ConsenSur II) n.d.:30. 20737129

[40] Sheth AN Althoff KN Brooks JT. Influenza susceptibility, severity, and shedding in HIV-infected adults: a review of the literature. Clin Infect Dis 2011; 52: 219–227. 21288848 10.1093/cid/ciq110 PMC4990828

[41] Coelho LE Ribeiro SR Veloso VG, et al Hospitalization rates, length of stay and in-hospital mortality in a cohort of HIV infected patients from Rio de Janeiro, Brazil. Braz J Infect Dis 2017; 21: 190–195. 27918889 10.1016/j.bjid.2016.10.007 PMC5489121

[42] Martínez E Marcos M Hoyo-Ulloa I, et al Influenza A H1N1 in HIV-infected adults*: influenza A H1N1 in HIV-positive adults. HIV Med 2011; 12: 236–245. 21255221 10.1111/j.1468-1293.2010.00905.x

[43] Peters PJ Skarbinski J Louie JK, et al HIV-infected hospitalized patients with 2009 pandemic influenza A (pH1N1)–United States, spring and summer 2009. Clin Infect Dis 2011; 52: S183–S188. 21342893 10.1093/cid/ciq036

[44] Ruuskanen O Jarvinen A. Editorial commentary: what is the real role of respiratory viruses in severe community-acquired pneumonia? Clin Infect Dis 2014; 59: 71–73. 24729504 10.1093/cid/ciu242 PMC4305147

